# Understanding immune checkpoint inhibitors-related myocarditis: Mechanisms and targeted therapeutic pathways

**DOI:** 10.7150/thno.127500

**Published:** 2026-06-17

**Authors:** Wenxiao Xia, Huan Zhang, Libo Liu, Chenchen Tu, Xiantao Song

**Affiliations:** Department of Cardiology, Beijing Anzhen Hospital, Capital Medical University, China.

**Keywords:** immune checkpoint inhibitors, myocarditis, cardiac autoimmunity, targeted therapies

## Abstract

Immune checkpoint inhibitor (ICI)-related myocarditis is a rare but often fatal adverse event that has gained increasing attention due to the widespread clinical use of ICIs. This review explores the development of immune cells and their critical roles in cardiac autoimmunity, with particular focus on defects in central tolerance, the role of immune checkpoints, T cell trafficking to the heart, and the involvement of resident cardiac macrophages. It also introduces the key pathogenic mechanisms of ICI-related myocarditis, including the activation of autoreactive T cells that recognize self or shared antigens, the crosstalk between T cells and macrophages, and macrophage polarization. In addition, we also discuss current and emerging targetable therapeutic pathways, including cytokine modulation, the JAK/STAT pathway, and the use of CTLA-4 immunoglobulin. We further summarize practical diagnostic and prognostic approaches, including the role and limitations of troponin-based monitoring, echocardiography, magnetic resonance imaging, PET-CT applications, and endomyocardial biopsy. This review aims to establish a mechanistic framework that integrates pathogenic mechanisms, diagnostic and prognostic approaches, and therapeutically targetable pathways in ICI-related myocarditis.

## Introduction

The development of immune checkpoint inhibitors (ICIs) has revolutionized cancer therapy, shifting the focus from conventional chemotherapy and radiotherapy to the promising realm of immunotherapy. By targeting key immune checkpoints, including cytotoxic T-lymphocyte antigen 4 (CTLA-4), programmed death-1 (PD-1), and programmed cell death 1 ligand 1 (PD-L1), ICIs reactivate T cell-mediated antitumor responses 1. Since the approval of the first anti-CTLA-4 agent for the treatment of advanced melanoma in 2011, ICIs have demonstrated remarkable clinical efficacy and an acceptable safety profile, effectively halting tumor progression and extending patient survival while further expanding their use in cancer treatment. However, in clinical practice, ICIs may induce a series of side effects known as immune-related adverse events (irAEs). The occurrence of irAEs increases the complexity of the treatment and deserves further attention. Reported cases of irAEs frequently include dermatological toxicities, colitis, hepatitis, pneumonitis, nephritis, and a range of endocrine disorders 2. Among these, the incidence of immune-related cardiac toxicity remains relatively low 3; however, fulminant ICI-related myocarditis, with grave complications, including cardiogenic shock, heart failure, and life-threatening arrhythmias, exhibits a significantly higher mortality rate than other irAEs, ranging from 25% to 50% 4,5. These adverse reactions highlight the balance between the therapeutic benefits of immunotherapy and its potential deleterious effects on cardiac function. In this review, we provide a spatiotemporal perspective on the intricate interplay between T cell development and cardiac autoimmunity. Subsequently, we discuss the distinct cellular compositions of cardiac macrophages and T lymphocytes, specifically highlighting their contributions to the maintenance of cardiac immune homeostasis. We further summarize practical diagnostic and prognostic approaches that combine biomarker surveillance with multimodal imaging in ICI-related myocarditis. This foundational context paves the way for a thorough exploration of the pathogenesis and targeted pathways for ICI-related myocarditis.

## 1. Critical roles of Macrophages and T Lymphocytes in Cardiac Autoimmunity

Healthy cardiac tissue harbors all major leukocyte populations, including monocytes/macrophages, neutrophils, B cells, and T cells 6,7. Lymphocytes account for 21%, and myeloid cells for 74% of cardiac immune cells, with macrophages representing the predominant myeloid population 8. However, in the steady state, the heart is not an immunologically active organ, and immune cells represent only a small fraction of cardiac cellularity. The distribution of these major cell types in the human heart has been explored using single-cell RNA sequencing (scRNA-seq), and differences between the atrial and ventricular tissues have been found^9^. Cardiomyocytes accounted for 30.1% of the atrial tissue, and immune cells (both myeloid and lymphoid) accounted for 10.4%. In contrast, the proportion of ventricular cardiomyocytes increased to 49.2%, whereas the proportion of immune cells decreased to 5.3% 9. However, the heart is not entirely immune-privileged. Its dense vascular and lymphatic networks enable immune cell trafficking and antibody access, and play crucial roles in pathogen clearance, inflammatory responses, limiting excessive immune damage, and immune tolerance 10. Here, we review the process of T cell development and its intersection with cardiac immunity, while also emphasizing the role of T cells and macrophages in cardiac immune homeostasis.

### 1.1 The Interplay of Spatiotemporal T Cell Development and Cardiac Immunity

#### 1.1.1 The Paradox of T Cell Central Tolerance and Cardiac Autoimmunity

**Figure 1:**
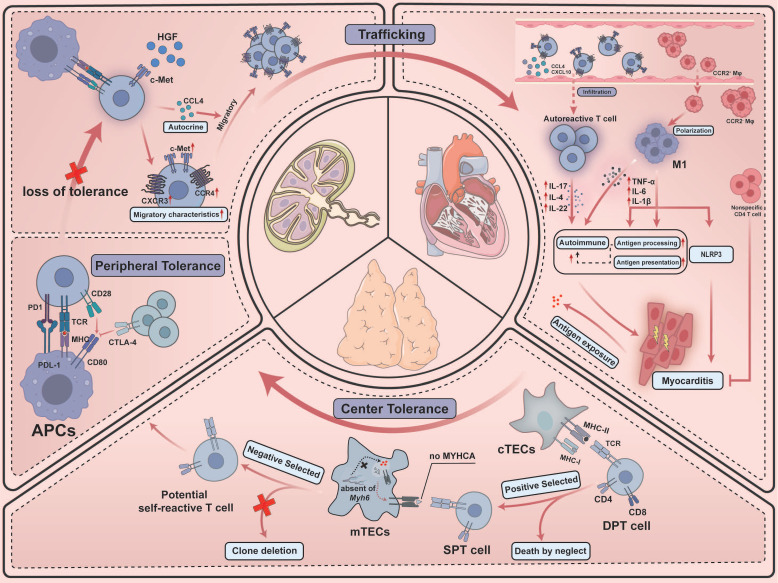
** Cardiac immune homeostasis and the fate of cardiac autoreactive T lymphocytes.** T cells undergo central tolerance in the thymus and then enter the periphery. Loss of peripheral tolerance confers upon them a migratory capacity, facilitating chemotactic recruitment to cardiac tissue and the initiation of an immune response. Specifically, double-positive thymocytes within the thymus, subjected to positive selection, differentiate into single-positive T cells. Given the absence within mTECs of genes encoding certain cardiac-specific tissue-restricted antigens (e.g., *Myh6*), potentially autoreactive T cells transit to the periphery. After exposure to cardiac-derived self-antigens and specific cardiac homing factors (e.g., HGF) following the failure of peripheral tolerance based on immune checkpoints for some reason, T cells acquire a cardiac trafficking label (e.g., c-Met^+^ CXCR3^+^ CCR4^+^) and infiltration into the myocardium, triggering an autoimmune inflammatory response in concert with macrophages.

T lymphocytes play a role in adaptive immune responses, which are essential for forming defense against infections and sustaining immune homeostasis. However, they can also initiate hypersensitivity reactions and autoimmune diseases. T cells originate in the bone marrow and subsequently migrate to the thymus, where they undergo central tolerance. Within the thymic cortex, immature CD4⁺CD8⁺ double-positive (DP) T cells interact with peptide-loaded major histocompatibility complex (pMHC) molecules presented by cortical thymic epithelial cells (cTECs) 11 **(Figure 1)**. DP T cells that fail to receive adequate T-cell receptor (TCR) signaling through these interactions undergo apoptosis by “death by neglect” within 3–4 days 12. Surviving thymocytes are positively selected to enforce MHC restriction and subsequently commit to CD4⁺ or CD8⁺ single-positive (SP) T cells based on the preferential recognition of MHC class II or class I, respectively 13. SP T cells then migrate into the medulla, where antigen-presenting cells (APCs) including medullary thymic epithelial cells (mTECs) and dendritic cells (DCs), implement negative selection to purge autoreactive TCR specificities 14. Promiscuous gene expression in mTECs enables them to express a substantial repertoire of tissue-restricted antigens (TRAs), typically found exclusively in specific tissues or cell types 15. Collectively, impairment of negative selection may allow autoreactive thymocytes to escape deletion and exit thymus, providing a mechanistic basis for autoimmune predisposition.

However, central tolerance exhibits an intrinsic vulnerability with respect to cardiac antigens, rendering the cardiac tissue particularly susceptible to autoimmune injury. Lv *et al.* discovered transcripts for *Myh6* (encoding α-myosin heavy chain, MYHCA) were absent from murine and human mTECs 16. Such distinct lapses in tolerance predispose the myocardium to autoimmunity, allowing latent populations of autoreactive T cells to persist in peripheral tissues until they are provoked by extrinsic stress. Specifically, the sterile inflammatory response to tissue necrosis mobilizes autoreactive T cells that evade thymic deletion. Borght *et al.* showed that myocardial infarction triggers cDC2 migration to draining lymph nodes, where presentation of MYHCA autoantigen promotes differentiation of quiescent CD4⁺ T cells into pathogenic Th1/Th17 effectors 17. Subsequent work by Forte *et al.* further revealed that the cDC1 subset orchestrates antigen cross-presentation through CLEC9A, a receptor that helps process antigens from necrotic cells and cross-priming of cytotoxic CD8^+^ T cells, thereby awakening latent autoreactive immune potential 18. Moreover, Cunha-Neto *et al.* identified CD4⁺ T-cell clones recognizing cardiac myosin in patients with Chagas disease and in corresponding animal models, consistent with a mechanism in which the *Trypanosoma cruzi*–derived B13 protein cross-reacts with MYHC epitopes and thereby activates latent MYHC-autoreactive CD4⁺ T cells 19. Notably, even in the absence of tissue damage, MYHCA can be constitutively presented to CD4^+^ T cells by cardiac APCs via MHC class II 17, and T cells derived from CTLA-4-deficient mice undergo vigorous spontaneous proliferation and precipitate severe ICI-related myocarditis 20 (see Section 3.1).

Taken together, cardiac TRA deficiency within mTECs presents a paradox: central T cell tolerance coexists with susceptibility to cardiac autoimmunity. Cardiac autoimmunity is fundamentally rooted in the intrinsic limitations of central tolerance. Thymic escape of cardiac-reactive T cells establishes a latent immunological substrate, whereas ischemic or infectious insults act as catalysts that convert this vulnerability into an overt pathology. This paradigm is further corroborated by the observation that alterations in the thymic epithelium, particularly in thymic epithelial tumors, are associated with both the incidence and severity of ICI-related myocarditis 21.

#### 1.1.2 T cell activation and trafficking to the heart

During the initiation of adaptive immune responses, naïve T cells encounter DCs within secondary lymphoid organs, where DCs present MHC molecules complexed with antigenic peptides 22. If TCRs specifically recognize these antigenic peptides and T cells simultaneously receive co-stimulatory signals, for example, through the interaction of CD28 with B7 family molecules (CD80, CD86), T cells initiate a specific transcriptional program that drives the functional differentiation of effectors, thus eliciting an immune response 23. CD4^+^ T cells release cytokines to attract and activate other immune cells, while CD8^+^ T cells gain cytotoxic abilities by directly eliminating the infected or neoplastic cells. Most effector T cells subsequently undergo apoptosis after pathogen clearance, while a subset persists as memory T cells 24. The surviving memory T cells constitute the predominant circulating population in the blood and are further classified into distinct subsets based on their phenotypes, functions, and migratory patterns: central memory T cells (Tcm, CD45RA^-^ CCR7^+^), effector memory T cells (Tem, CD45RA^-^ CCR7^-^), and stem cell memory T cells (Tscm, CD45RA^+^ CCR7^+^ CD95^+^ CD122^+^) 25,26. This classification method may be flawed, however, because there may be overlap between different subpopulations and cells within the same subpopulation may have different functional properties 27. Moreover, although the majority of effector T cells succumb to apoptosis following infection, it has been observed in humans that a population of terminal effector cells displaying the CD45RA^+^ CCR7^-^ phenotype, known as Temra cells, can persist within the circulation 28. Temra cells represent a highly differentiated subset within the human CD8^+^ memory T cell lineage, and they increase with age 28. While possessing a limited proliferative capacity, Temra cells can produce significant amounts of IFN-γ and IL-2, as well as express elevated levels of perforin and FasL, endowing them with potent cytotoxic capabilities 29.

Defining the determinants of organ-specific T cell trafficking, including the heart, remains challenging. Distinct memory T cell subsets exhibit organ-specific homing capabilities, interacting with the endothelial cells (ECs) of their respective target tissues, and soluble factors secreted by the tissues themselves may contribute to the directed recruitment of these T cell populations 30. Specifically, within the lymph nodes **(Figure 1)**, hepatocyte growth factor (HGF) derived from the heart binds to the c-Met receptor on T cells, thereby inducing memory T cells to acquire distinctive migratory characteristics (c-Met^+^ CCR4^+^ CXCR3^+^) that are specific to the heart 31. Furthermore, c-Met signaling promotes the autonomous release of chemokines (e.g., CCL4), thereby establishing an autocrine chemokine loop that further enhances the migration of T cells toward the heart 31. This increase in circulating c-Met^+^ memory T cells may delineate the characteristics of adaptive cardiac inflammation, and the elevation of autoantigen-specific c-Met^+^ T cells in peripheral blood marks the loss of cardiac immune tolerance 32. In humans, c-Met^+^ T cells infiltrate inflamed myocardium in acute myocarditis and idiopathic dilated cardiomyopathy, respond to the cardiac myosin and secrete cytokines such as IL-4, IL-17, and IL-22 32. In addition, scRNA-seq analysis of cardiac CD45^+^ cells showed that cytotoxic T cells were the largest T cell population in the normal heart, and Th17 cells appeared in large numbers in the early stages of cardiac autoimmune acute inflammation and reached a peak at day 14 33. In later stages, myocardial non-specific CD4^+^ T cells were effective in inhibiting post-inflammatory fibrotic remodeling 34.

In effector T cell-mediated immune responses, antigen-specific activated lymphocytes must be efficiently localized to antigen-rich nonlymphoid tissues, and the HGF-c-Met signaling pathway defines the homing of memory T cells toward the heart 32. The balance among T cell populations functions as a double-edged sword; however, the precise roles and mechanisms of various cell subsets remain a subject of debate 33,34. Defining the molecular determinants of cardiac homing and resolving the functional heterogeneity among cardiac-infiltrating T cells remains a priority.

### 1.2 Diverse Macrophage Subsets in Cardiac Immune Homeostasis and Autoimmunity

Resident cardiac macrophages (RCMs) are the most abundant immune cells in the adult heart, accounting for 6% to 10% of non-cardiomyocytes 35. Myocardium contains distinct RCM subsets classified by their divergent origins and marker expression 36: Monocytes in the circulatory system can express CCR2, a chemokine receptor crucial for migration 37, and these monocytes can infiltrate locally into affected tissues, replenishing RCM populations and contributing to the progression of cardiovascular disease 38. In contrast, CCR2^-^ RCMs originate from embryonic progenitor cells in the yolk sac and maintain the mostly resident population of cardiac tissue at homeostasis by localized proliferation 39. Upon myocardial injury, RCMs populations expand, in part because CCR2^+^ RCMs release MCPs to recruit additional monocytes via a MYD88-dependent mechanism, rather than enhancing ECs adhesion or transendothelial migration 40. As immune profiling has advanced, additional macrophage subsets have been identified. Based on common life cycle properties and core gene signatures in mouse hearts, scRNA-seq identifies three distinct subsets of macrophages: TLF^+^ macrophages (TIMD4^+^ LYVE1^+^ FOLR2^+^ MHC-II^lo^ CCR2^-^) are sustained independent of blood monocytes; MHC-II^hi^ macrophages (TIMD4^-^LYVE1^-^ FOLR2^-^ MHC-II^hi^ CCR2^-^) are partially replaced by monocytes; and CCR2^+^ macrophage subsets (TIMD4^-^LYVE1^-^FOLR2^-^ MHC-II^hi^ CCR2^+^) are entirely substituted by monocytes 41,42. Across these lineages, tissue niches shape macrophage programs via cues such as IL-34 and M-CSF 43,44, consistent with expression of *CD163*, *COLEC12*, *MRC1*, *MARCH1*, and *NRAMP1* 6.

Macrophages also dominate autoimmune myocarditis 33. Infiltrating cardiac monocytes can differentiate into M1-type macrophages **(Figure 1)**, which release pro-inflammatory cytokines such as TNF-α, IL-6, and IL-1β, thereby precipitating tissue damage and an inflammatory cascade 45. In experimental autoimmune myocarditis (EAM), acute inflammation features an inflammation-associated macrophage cluster enriched for *Hif1a*-related genes and nitric oxide production, supporting enhanced antigen processing and endogenous peptide presentation 33. Hua *et al.* demonstrated that the STING inhibitor C-176 alleviates myocardial inflammation and functional impairment in EAM mice, affirming STING's role in enhancing pro-inflammatory macrophage polarization via the HIF1α pathway and thereby contributing to the pathogenesis of autoimmune myocarditis 46. Furthermore, macrophages engage in the NLRP3 inflammasome-mediated pyroptosis that exacerbates EAM progression through the PPARα/LACC1/NF-κB signaling cascade 47.

In the preclinical ICI-related myocarditis model using *Ctla4*⁺^/^⁻*Pdcd1⁻^/^⁻* mice, GO enrichment analysis indicates that CCR2⁻ CD163⁺ cardiac macrophages are preferentially engaged in regulating epithelial cell proliferation as well as in orchestrating stress responses 48. This profile mirrors the role of CCR2⁻ RCMs as a homeostatic subpopulation essential for tissue maintenance and anti-inflammatory regulation 49. Consistently, in endomyocardial biopsy specimens from patients with ICI-related myocarditis, CCR2⁻ macrophages defined by CD163, LYVE1 and FOLR2 expression retain homeostatic and tissue-support programs without evidence of inflammatory reprogramming 50. By contrast, in *Ctla4⁺^/^⁻Pdcd1⁻^/^⁻* mice, an IFN-γ–inducible Cxcl9⁺Cxcl10⁺ macrophage subset has been identified, characterized by chemokine production and enhanced antigen-presenting capacity 48. Trajectory analysis, together with CCR2 blockade using the MC-21 antibody, indicates that these cells arise from CCR2⁺ monocyte-derived macrophages 48. In ICI-related myocarditis, CCR2⁺ macrophages thus acquire IFN-γ–driven antigen-presenting and chemokine-producing programs and undergo selective expansion 51, establishing CCR2⁺ macrophages as a central pathogenic population that amplifies myocardial injury through the CXCL9/CXCL10–CXCR3 axis (see also Section 3.2.1).

## 2. The Emergence of Cardiotoxicity Associated with Immune Checkpoint Inhibitors

### 2.1 The Multifaceted Roles of Immune Checkpoints in Cardiac Autoimmunity

Examples of α-myosin–reactive T cells underscore that not all cardiac TRAs are represented in the thymus 16, permitting thymic escape of autoreactive clones. Peripheral tolerance therefore becomes essential, and immune checkpoints constitute a central inhibitory layer within this system. As inhibitory receptor–ligand pairs on immune cells, CTLA-4 and PD-1/PD-L1 function as core “brakes” that set activation thresholds for T cells and help preserve cardiac immune homeostasis.

#### 2.1.1 CTLA-4

CTLA-4 and CD28 are homologous receptors expressed on both CD4^+^ and CD8^+^ T cells 52. CTLA-4, with a higher affinity for the CD28 ligands CD80 and CD86, outcompetes CD28 for binding, thereby blocking co-stimulatory signaling and inducing T cell anergy, a state where T cells, despite recognizing an antigen, fail to become fully activated and proliferate 52. Furthermore, CTLA-4 can physically limit the availability of costimulatory molecules. Emerging research indicates that CTLA-4 can engage in trans-endocytosis, which directly captures CD80 and CD86 from the surface of APCs and internalizes them for degradation 53. This mechanism directly reduces the costimulatory capacity of APCs, providing an additional layer of immune regulation 54. However, the trans-endocytosis model requires further investigation to fully elucidate its intricacies and implications.

The immunomodulatory role of CTLA-4 in the heart is similar to its role in other tissues. It inhibits T-cell activation and proliferation, thereby maintaining homeostasis and reducing autoimmune attacks in cardiac tissues 55. Congenital CTLA-4-deficient mice develop fatal myocarditis, whereas adult-deficient mice do not have significant, death-inducing cardiac pathologic alterations, suggesting that the role of CTLA-4 in cardiac immune tolerance may be related to the developmental period 56. Furthermore, CTLA-4 plays a key role in the maintenance of allograft tolerance by reducing rejection of the transplanted heart and affecting long-term graft survival by inhibiting T-cell activation 57.

#### 2.1.2 PD-1/PD-L1

PD-1 is an inhibitory receptor expressed on the surface of T cells that engages with its ligands, PD-L1 and PD-L2, which are primarily presented by APCs and other cell types. PD-L1 is a ubiquitous ligand that is widely expressed across multiple cells, including T cells, B cells, APCs, vascular and stromal endothelial cells, and pancreatic islet cells 58,59, whereas the expression of PD-L2, a ligand predominantly expressed by DCs, macrophages, and B cells, is more restricted 58,59. Unlike CTLA-4, PD-1 primarily suppresses T cell activation through signal transduction pathways. Mechanistically, PD-1 inhibits Akt phosphorylation by blocking CD28-mediated PI3K activation, thereby downregulating transcriptional programs responsible for T cell activation, proliferation, and survival 60. During the effector phase of the immune response, the absence of PD-1 expression can lead to an increase in T cell proliferation or specific effector functions 61. Moreover, PD-1 deficiency also skews CD8^+^ T cells towards a Tcm phenotype, characterized by elevated expression levels of CD62L, CD27, and CCR7, and enhanced IL-2 production 62.

**Figure 2:**
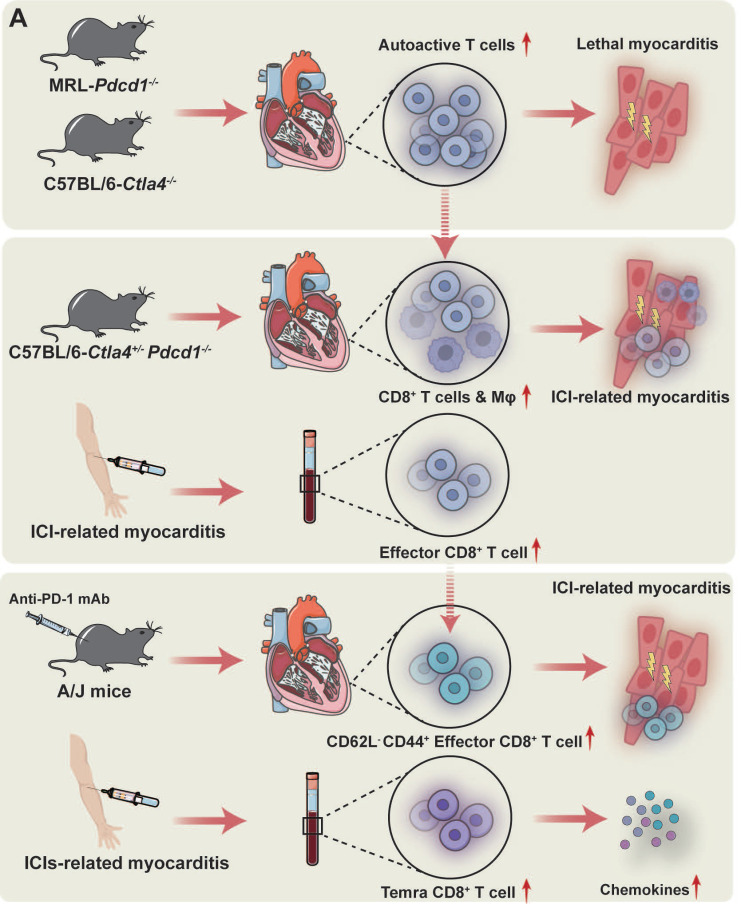

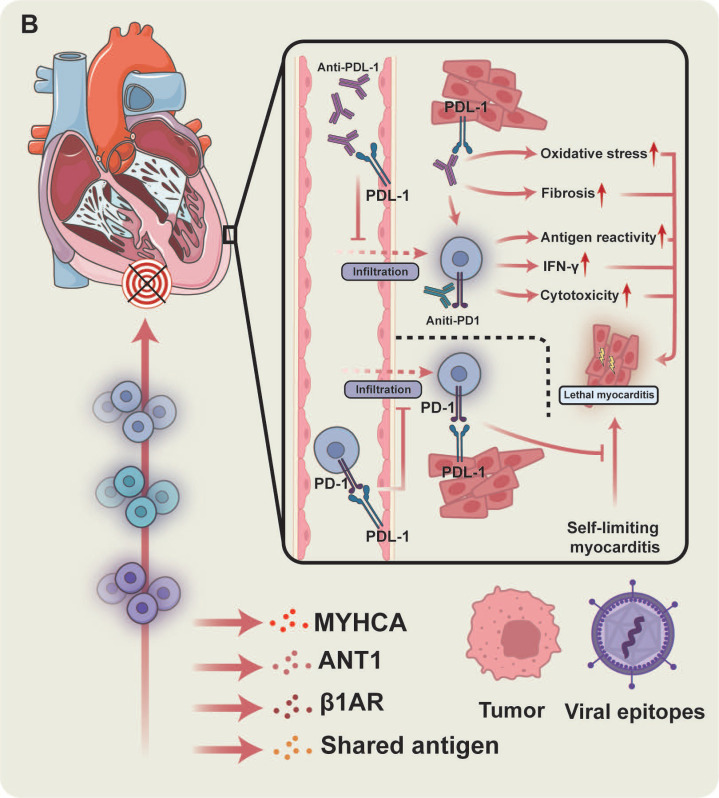
** The primary pathogenic mechanisms underlying ICI-related myocarditis.** (A) Exacerbated autoreactive CD8^+^ T cell activity is a key driver in ICI-associated myocarditis and insight into the precise pathogenic T cell subsets is progressively clarifying. (B) MYHCA is a primary target antigen for cardiac T cell attack, though additional autoantigens, or antigens shared by tumors and viruses, potentially contribute. The presented figure demonstrates a protective role for PD-1/PD-L1 within the heart and the detrimental consequences of disrupting this axis.

PD-L1 is expressed in the heart, and markedly elevated levels of PD-L1 mRNA have been detected in mouse cardiac tissue 63. *PD-L1* expression induced on cardiac ECs plays a critical immunomodulatory role **(Figure 2B)**. Endothelial cells activated by IFN-γ inhibit T cell activation through the expression of PD-L1 and PD-L2, and blocking PD-L1 with antibodies enhances the costimulatory effects of endothelial cells on CD8^+^ T cells, amplifying their responsiveness to antigen presentation by ECs, which includes increased IFN-γ secretion and augmented cytotoxic activity 64. Consistent with this, studies involving genetically modified mice lacking PD-L1 and PD-L2 have demonstrated that the induced expression of PD-L1 on cardiac ECs plays a crucial role in immune modulation within the heart. This mechanism can safeguard cardiac tissue from damage inflicted by CD8^+^ T cells and the inflammation induced by polymorphonuclear leukocyte-rich microabscesses, thus preventing otherwise self-limiting myocarditis from progressing into a life-threatening condition 65. Moreover, the inflammation and oxidative stress resulting from PD-1 deficiency may precipitate electrical and structural remodeling of the atria, thereby increasing the risk of atrial fibrillation 66. In neonatal mice, PD-1 deficiency exacerbates the inflammatory response following cardiac injury, as evidenced by increased monocyte infiltration, heightened expression of pro-inflammatory factors, and worsened fibrosis 67. Concurrently, DN T cells in these mice display heightened cytotoxic sensitivity to cardiac antigens, underscoring the critical role of the PD-1/PD-L1 pathway in cardiac protection and regeneration by inhibiting inflammation and autoimmune damage 67. Although PD-L1 has been extensively studied in the context of cardiac immune homeostasis, the specific role of PD-L2 remains to be elucidated. In EAM models, PD-L2 deficiency is associated with exacerbated myocardial inflammation, and flow cytometric analyses have revealed a significant increase in inflammatory cells 68. Moreover, DCs lacking PD-L2 exhibited enhanced proliferation of CD4^+^ T cells in response to TCR and CD28 signaling, suggesting that PD-L2 may play a critical role in curbing the expansion of autoreactive CD4^+^ T cells and subsequent inflammation 68.

### 2.2 The Application of Immune Checkpoint Inhibitors and Related Myocarditis

Immune checkpoints are essential for maintaining immune homeostasis and preventing immune system hyperactivity by promoting peripheral tolerance mechanisms. However, tumor cells can exploit these checkpoint mechanisms to evade immune surveillance, thereby enhancing their survival and proliferation 69. Specifically, tumor cells induce immune resistance and T cell exhaustion by expressing checkpoint proteins such as PD-L1, which inhibit T cell activation and proliferation through the engagement of these inhibitory molecules 69. Given that these checkpoints can be readily blocked by antibodies or regulated through the reconfiguration of ligands and receptors 70, ICIs targeting proteins, such as CTLA-4, PD-1, and PD-L1 have been developed to relieve the immune suppression and reactivate T cell-mediated immune responses, thereby inducing attacks against tumors 1.

ICIs therapy has fundamentally transformed the field of oncology. Since the approval of the first anti-CTLA-4 agent for advanced melanoma in 2011, ICIs have heralded a new era of cancer treatment. Subsequently, various ICIs have been approved for the treatment of melanoma, lung cancer, colorectal cancer, hepatocellular carcinoma, and renal cell carcinoma have been approved for clinical use, offering patients novel therapeutic alternatives 71-74. These ICIs include anti-CTLA-4 monoclonal antibodies (mAbs) such as ipilimumab, anti-PD-1 mAbs (pembrolizumab, nivolumab, and cemiplimab), anti-PD-L1 mAbs (atezolizumab, avelumab, and durvalumab), and anti-LAG-3 mAbs. Collectively, they exhibit remarkable clinical efficacy and an acceptable safety profile, effectively inhibiting tumor progression and prolonging survival, while their indications continue to expand.

Despite their efficacy, ICI-induced immune activation can lead to a range of side effects known as irAEs. In addition to issues related to drug resistance that limit the number of patients who can achieve sustained benefits 75, the occurrence of irAEs adds complexity to treatment and warrants increasing attention. In cancer patients receiving ICIs, reported cases of irAEs commonly include skin toxicity, colitis, hepatitis, pneumonitis, nephritis, and endocrine disorders, such as thyroid dysfunction 2.

Recently, cardiac toxicity has emerged as a major concern. Although immune-related cardiac toxicity is less frequent than other irAEs, the estimated one-year risk of developing pericarditis or myocarditis is 1.8% 3. However, due to its potential to lead to severe outcomes such as cardiogenic shock, heart failure, and fatal arrhythmias, the associated mortality rate is significantly higher, ranging from 25% to 50% compared to other irAEs 4,5. Additionally, an analysis of the burden of cardiac disease toxicity associated with ICIs in cancer patients aged 65-85 years indicates that cardiac disease is one of the primary disease types resulting from these ICIs 76. This underscores the importance of early recognition and management of immune-related cardiac toxicity. Pharmacovigilance analysis of ICIs has identified potential ICI-related major cardiac adverse drug reactions, including myocarditis, atrial fibrillation, heart failure, pericardial effusion, tachycardia, myocardial infarction, sinus tachycardia, acute myocardial infarction, pericarditis, and cardiac tamponade 5,77. Among fatal ICI-related cardiac adverse drug reactions, myocarditis is the most common, accounting for 50.8% of cases, making it a crucial focus in ICI cardiotoxicity research 5.

### 2.3 Temporal Characteristics of Myocarditis Onset

The onset of myocarditis after ICIs therapy exhibits significant individual variability. Most patients develop symptoms during the early stages of treatment, with the earliest manifestations potentially occurring after the first administration of ICI therapy. Specifically, Mahmood *et al.* conducted a retrospective case-control study and found that the median time to the onset of myocarditis after the initiation of ICIs therapy was 34 days, with 81% of cases occurring within three months of treatment initiation 4. Similarly, a pharmacovigilance study of cases in the VigiBase database reported a median time to onset of 33 days (interquartile range: 21–91 days) of myocarditis symptoms after the first administration of ICIs therapy 77. However, a small cohort of patients, presents with delayed-onset ICI-related myocarditis, which manifests several months or even longer after the commencement of treatment. In a notable report by Yamaguchi *et al.*, a case of late-onset fulminant myocarditis was observed after 13 cycles of nivolumab therapy 78. Furthermore, delayed immune-related events following ICI discontinuation are gaining recognition. Nguyen *et al.* reported the longest latency period yet for ICI-related myocarditis, with a 33-week interval between pembrolizumab cessation for metastatic colorectal cancer and myocarditis diagnosis, exceeding two years from the initiation of treatment 79. These findings underscore the surveillance of immune-related cardiac toxicity. The diagnosis of ICI-related myocarditis and meticulous monitoring of the cardiac status should not be confined solely to the early phases of ICI treatment; rather, vigilance must be maintained, even during prolonged therapy and after the cessation of treatment.

### 2.4 Multidrug Combinations as Principal Risk Factors

Monotherapy with PD-1/PD-L1 inhibitors has been linked to an increased risk of cardiovascular adverse events (CVAEs) across all severity grades^80^. Notably, treatment groups receiving anti-PD-1 antibodies demonstrate a higher incidence of cardiac adverse events (69.4%) compared to those treated with anti-CTLA-4 antibodies (20%) 5. Among all therapeutic regimens, combined PD-L1 and CTLA-4 blockade carries the highest risk of CVAEs of any grade 81. The analysis of the VigiBase pharmacovigilance database corroborates this observation, indicating that combination ICI therapy is associated with a higher mortality rate for myocarditis compared to ICI monotherapy 77.

Klocke *et al.* demonstrated that thymic escape of CTLA-4–deficient autoreactive clonal T cells establishes a foundational immunological substrate for myocarditis susceptibility 56. In parallel, Wei *et al.* revealed a gene dosage–dependent functional interplay between CTLA-4 and PD-1, whereby *Ctla4* haploinsufficiency combined with *Pdcd1* loss precipitates fulminant ICI-related myocarditis 82. Furthermore, genetic or pharmacologic dual checkpoint disruption accelerates CD8⁺ T-cell and CCR2⁺ macrophage myocardial infiltration and activates the CXCL9/CXCL10–CXCR3 axis, establishing a feed-forward loop in myocarditis 51. Collectively, CTLA-4 and PD-1 operate at complementary checkpoints of the T-cell response, with CTLA-4 constraining priming and costimulation in lymphoid organs and PD-1 limiting effector activity in peripheral tissues, including the heart. Beyond emphasizing the need for intensified baseline counseling and early surveillance with dual regimens, these mechanistic insights provide a rationale for restoring co-inhibitory signaling using CTLA-4-Ig in steroid-refractory ICI-related myocarditis (see Section 3.5).

## 3. The Immunological Mechanisms and Potential Therapeutic Targets in ICI-Related Myocarditis

### 3.1 The Excessive Activation of Autoreactive T Cells is Central to the Pathogenesis of ICI-related Myocarditis

#### 3.1.1 Autoreactive CD8^+^ T Cells

Preclinical models and human data converge on the critical role of T cells in the pathogenesis of ICI-related myocarditis, highlighting the overactivation of autoreactive T cells as a necessary condition for its development. The MRL-*Pdcd1*^-/-^ mouse model spontaneously develops lethal myocarditis, characterized by a massive infiltration of lymphocytes and myeloid cells within cardiac tissue 83. Adoptive transfer experiments further support the notion that PD-1 deficiency leads to the proliferation and activation of autoreactive T cells 83. Mice deficient in *Ctla4* exhibit a phenotype analogous to that of *Pdcd1*-deficient mice, albeit with greater severity, characterized by persistent T cell activation, infiltration across multiple organs, and subsequent tissue damage, ultimately leading to premature mortality 20.

Wei and colleagues established a C57BL/6 background model featuring *Ctla4^+/-^* and *Pdcd1^-/-^* strains, whereby premature mortality was attributed to cardiac T cell and macrophage infiltration, alongside significant electrocardiographic abnormalities, mirroring the clinical and pathological features of ICI-related myocarditis observed in patients 82. Notably, this study validated that the gene dosage of *Ctla4* and *Pdcd1* collectively dictates the threshold for T cell self-recognition, with their functional interaction deemed critical for maintaining cardiovascular homeostasis 82.

Further characterization of the T cell phenotype revealed a substantial presence of highly activated and proliferating, clonal CD8^+^ T cells within the myocardium of* Pdcd1^-/-^Ctla4^+/-^* mice 82,84. Adoptive transfer of immune cells from myocarditis mice into *Rag1*^-/-^ recipients induced lethal myocarditis; conversely, CD8^+^ T cells depletion abrogated this induction 84. Consistent with these findings, scRNA-seq of peripheral immune cells from patients with PD-1 inhibitor-related myocarditis revealed intense peripheral immune responses, specifically an increased proportion of CD8^+^ T cells 85. Analogous findings have been observed in an inducible myocarditis pharmacological model. Administration of anti-PD-1 mAb to A/J mice successfully induced ICI-related myocarditis, resulting in robust activation of autoreactive CD8^+^ T cells 86. Specifically, a significant elevation in the proportion of CD62L^-^CD44^+^ effector T cell subsets was observed within the myocardium of the affected mice, whereas the proportion of CD62L^+^CD44^+^ memory T cell subsets remained comparable to controls 86. A comprehensive analysis of peripheral blood mononuclear cells (PBMCs) from patients with ICI-related myocarditis, utilizing CyTOF, scRNA-seq, and TCR-seq, revealed a significant expansion of Temra CD8^+^ T cells in the peripheral blood 87. These Temra CD8^+^ T cells exhibit pronounced activation and cytotoxic potential, while the frequencies of other major circulating immune populations were largely unchanged 87. Notably, these cells demonstrate elevated expression of chemokines CCL5, CCL4, and CCL4L2, potentially promoting cardiac T cells migration and contributing to the pathogenesis of ICI-related myocarditis 87. Recent investigations have implicated a protective role for CD4^+^ TCM cells in ICI-related myocarditis injury, and their proportion is inversely correlated with myocarditis severity, which stems from the elevated expression of immunosuppressive factors such as IL-4I1, effectively mitigating the proliferation and activation of Temra CD8^+^ T cells 88. Further studies are needed to elucidate the precise mechanisms underlying the protective function of CD4^+^ TCM cells in ICI-related myocarditis.

#### 3.1.2 Cardiac Self-antigens and Shared Antigens as a Key to T Cell-mediated Attacks on the Heart

What initiates the transition from autoreactive potential to overt myocardial injury remains an active question. The prevailing hypotheses posit that cardiac-specific proteins, particularly MYHCA, are pivotal in driving effector T cells toward an autoimmune assault on the heart in patients with ICI-related myocarditis. Previous sections have delineated the inherent vulnerability of cardiac immune tolerance mechanisms, specifically, the insufficient expression of MYHCA in mTECs. This deficiency prevents the deletion of α-myosin-reactive T cells within the thymus, allowing their release into the peripheral circulation. In *Pdcd1*^-/-^*Ctla4*^+/-^ mice, analysis of the TCR repertoire identified α-myosin as the target antigen for three dominant clonal TCRs in this model of ICI-related myocarditis 84. Further investigation revealed the presence of α-myosin-specific TCRs in blood samples from ICI-related myocarditis patients, alongside significant clonal expansion of these TCRs in both the cardiac and skeletal muscles of these patients 84. Moreover, the treatment of A/J mice with anti-PD-1 antibodies may reactivate previously quiescent MYHCA-specific T cells and potentially precipitate myocarditis 86. Recent findings by Kalinoski *et al.* demonstrated the presence of autoreactive MYHCA tissue resident memory (TRM) cells co-expressing PD-1 and CD69 within the healthy heart; during post-injury cardiac recovery stages, these TRM cells accumulate within the epicardium, which may increase susceptibility to ICI-related cardiomyopathy 89.

Beyond MYHCA, additional cardiac self-antigens have been proposed. Employing A/J mice bearing distinct adenine nucleotide translocase 1 (ANT1) peptide sequences, Basavalingappa *et al.* demonstrated that the mitochondrial protein ANT1 can induce EAM, with the ANT1 21-40 peptide fragment identified as the principal myocarditis epitope, and associated with IL-17A production by T cells 90. Immunization of A/J mice with specific β1AR peptide sequences revealed their ability to bind major histocompatibility complex class II/IAk or IEk alleles, thereby inducing varying degrees of myocarditis 91. This β1AR peptide-mediated response further demonstrated an enhanced clonal expansion of TCRs 92.

Tumor cells may express antigens homologous or identical to those found in normal tissues, potentially leading to autoimmune toxicity against healthy tissues during the immune system's recognition and attack of tumor cells. This cross-reactivity, which induces autoreactive T cells, has been implicated in lung irAEs 93. Case reports have documented the frequent presence of shared T cell receptor sequences in cardiac, skeletal muscle, and tumor tissues. The elevated expression of muscle-specific transcripts within tumor tissues further supports the possibility of cross-reactivity with autoantigens, including *Myh6* 94. Analysis of the TCGA melanoma cohort reveals detectable *Myh6* expression in 69% of cases 84. However, approximately 40% of melanoma tumor cells aberrantly express *Myh6* in ICI-treated patients without clinically significant myocarditis or myositis 84. A case study of three patients developing severe myocarditis and myositis within three weeks of both COVID-19 booster vaccination and PD-1 blockade therapy revealed overlapping T cell receptor repertoires, potentially due to cross-reactivity between pre-existing T cell responses to viral epitopes (e.g., the spike protein) elicited by mRNA COVID-19 vaccines and muscle antigens 95. Although cross-reactivity between tumor and autoantigens does not appear to be a determinant factor in ICI-related myocarditis, further investigations are necessary to uncover potential yet-unrecognized cross-reactive pathways.

In summary, excessive activation of autoreactive T cells serves as a critical prerequisite for ICI-related myocarditis. Both animal models and human data indicate that deficiencies in PD-1 or CTLA-4 lead to the proliferation and activation of autoreactive T cells, ultimately culminating in cardiac inflammation and tissue damage** (Figure 2A)**. Cardiac-specific proteins, particularly MYHCA, are considered key elicitors of T cell-mediated attacks on the heart, although contributions from other self-antigens and the possibility of epitope spreading from tumors should also be considered** (Figure 2B)**.

### 3.2 Macrophages: an Indispensable Component

#### 3.2.1 The CXCL9/CXCL10-CXCR3 Axis

Given the characteristic macrophage infiltration observed in ICI-related myocarditis, the interplay between T cells and macrophages has received heightened scrutiny in understanding the pathogenesis of ICI-related myocarditis. In the *Ctla4*^+/-^*Pdcd1*^-/-^ mice, Pan *et al.* identified a CXCL9^+^CXCL10^+^ CCR2^+^ macrophage subset within cardiac tissue 48. These macrophages are derived from circulating CCR2^+^ monocytes and are shaped by T cell-derived IFN-γ; a similar macrophage subpopulation has also been observed in human cases of ICI-related myocarditis 48. CD8^+^ T cells depletion or the blockade of the IFN-γ signaling pathway resulted in a reduction of CXCL9^+^CXCL10^+^ macrophages and an amelioration of myocarditis symptoms in murine models 48. Huang *et al.* demonstrated the accumulation of CXCL9/10^+^ CCR2^+^ macrophages and clonally expanded CXCR3^hi^CD8^+^ T cells in the MRL-*Pdcd1*^-/-^ model 51. By selectively targeting and disrupting the interactions between these T cells and the CXCL9/10 released by macrophages, the research prevented and treated ICI-related myocarditis in the model 51. This finding further supports the notion that the IFN-γ signaling pathway, along with the CXCL9/CXCL10-CXCR3 axis, mediates a positive feedback loop between T cells and macrophages, thereby exacerbating cardiac inflammation and injury.

#### 3.2.2 Macrophage Polarization Intensifies Myocarditis

PD-1 inhibitors are associated with M1 polarization of cardiac macrophages and increased expression of pro-inflammatory mediators, including iNOS, IL-1β, IL-6, and TNF-α, consistent with amplified inflammatory responses and myocardial injury 96. Mechanistically, TargetScan analysis and luciferase assays demonstrate that miR-34a targets the 3' untranslated region of KLF4 96. Inhibition of miR-34a or overexpression of KLF4 effectively reverses PD-1 inhibitor-induced M1 polarization and cardiac damage, suggesting that KLF4 exerts a protective anti-inflammatory effect, whereas miR-34a promotes cardiac injury by suppressing KLF4 in the heart. 96. In addition, macrophages can affect the cell cycle and telomere length through the accumulation of miR-34a-5p in exosomes and its translocation into cardiomyocytes, which in turn targets the 3'-untranslated region of the serine/threonine protein phosphatase 1 regulatory subunit 10 (PNUTS) of cardiomyocytes, leading to cardiac senescence and dysfunction 97. These findings provide novel insights into the mechanisms underlying ICI-related cardiac injury, underscoring the need for further investigations to substantiate the relationship between miR-34a and immunotherapy-induced cardiotoxicity.

Antibodies targeting CTLA-4 m2a may exacerbate cardiac injury in EAM mice by promoting the infiltration of neutrophils, particularly a subset of Ccl5-expressing neutrophils, which in turn drives macrophages toward a pro-inflammatory M1 phenotype, resulting in overall cardiac inflammation, fibrosis, and dysfunction 98. Extracellular vesicles derived from human bone marrow mesenchymal stem cells (hBMSC-Exos) can mitigate cardiac damage in melanoma mice induced by BMS-1 (PD-1/PD-L1 inhibitor). These hBMSC-Exos inhibit the M1 macrophage polarization induced by BMS-1 while promoting a shift toward M2 macrophage polarization, accompanied by reduced expression levels of pyroptosis-related proteins such as ASC, caspase-1, NLRP3, and GSDMD 99.

**Figure 3:**
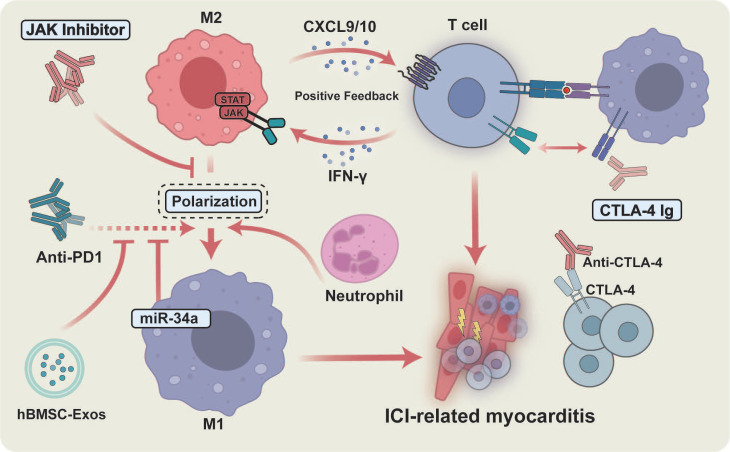
** Therapeutic interventions for ICI-related myocarditis focus on modulating the interplay between CD8^+^ T cells and macrophages, specifically M1 polarization.** Positive feedback loops driven by the IFN-γ/JAK/STAT1 and CXCL9/10/CXCR3 axes, promoting T cell–macrophage synergy and M1 macrophage polarization, amplify immune-mediated inflammation and tissue damage in ICI-related myocarditis. JAK inhibition represents a promising novel therapeutic avenue targeting this mechanism.

In summary, by modulating the interplay between CD8^+^ T cells and macrophages **(Figure 3)**, as well as the polarization of macrophages—particularly through the inhibition of M1 macrophage polarization, it is possible to effectively attenuate the inflammatory response and damage associated with myocarditis, thereby offering novel insights and potential therapeutic targets for the treatment of this condition.

### 3.3 Exploratory Cytokine Targeting Pathways for ICI-related Myocarditis

Building on the pathogenic framework outlined above, in which autoreactive T cells and inflammatory macrophages form a self-reinforcing circuit, cytokines can be viewed as network-level amplifiers that couple cellular crosstalk to tissue injury. In ICI-related myocarditis models, macrophage-derived cues (e.g., IL-6 and IL-23) promote effector skewing and expansion of pathogenic CD4⁺ T-cell states 100,101, whereas T-cell–derived cytokines (notably IL-17A) act downstream to potentiate myocardial inflammation 102, remodeling and functional decline. Importantly, these mediators are not introduced here as isolated biomarkers but represent intervenable targets that mechanistically connect the previously described T-cell–macrophage interactions with organ-level cardiotoxicity, while offering a rational opportunity to decouple antitumor efficacy from ICI-related toxicity.

In a preclinical pharmacological model of PD-1 blockade, CD4⁺ T cells are skewed towards Th17 differentiation, thereby emerging as the predominant source of IL-17A 103. This shift is mirrored by increased Th17 infiltration of the myocardium, coupled with upregulated *Il17a* and RORγt expression and enhanced IL-17A staining within cardiac tissue 103. Consistently, in two cases of ICI-related myocarditis, IL-17 staining of endomyocardial biopsy specimens suggested myocardial Th17 infiltration 104. In the TnI–induced autoimmune myocarditis (TnI-AM) mouse model, Bockstahler *et al.* showed that cytokines from CD11b⁺ monocyte–derived cells, particularly IL-6, not only amplify inflammation but also expand autoreactive CD4⁺ T cells and bias their differentiation toward a Th17 phenotype, which is associated with more severe cardiac inflammation and fibrosis 104. Genetic deletion of the immunoproteasome component LMP7 or pharmacological inhibition with ONX 0914 markedly reduced cardiac IL-17 expression and alleviated myocardial inflammation 104. Importantly, the TnI-AM model does not recapitulate ICI-related myocarditis. In contrast, in murine models of ICI-related myocarditis, the upstream cues that commit CD4⁺ T cells to Th17 remain incompletely defined. What is clearer from available intervention studies is that Th17-derived IL-17A acts as a key downstream inflammatory mediator of ICI-related cardiotoxicity, aligning with adverse cardiac remodeling, fibrosis, and a heart failure–like functional decline 102. In PD-1 blockade models, heightened IL-17 signaling accompanies cardiac dysfunction, whereas IL-17A neutralization (or CD4⁺ T-cell depletion) can prevent deterioration in cardiac performance 102.

IL-6 is a crucial cytokine in the differentiation of naïve CD4^+^ T cells into Th17 cells, a process that is intricately linked to the pathogenesis of various autoimmune diseases. In addition to the aforementioned findings in the TnI-AM mouse model 104, IL-6 released by CD11b⁺ macrophages drives CD4⁺ T cell differentiation towards a Th17 phenotype, which is closely associated with exacerbated cardiac inflammation and fibrosis 105. Chen *et al.* established a lung cancer mouse model of PD-1 inhibitor–induced myocarditis and, by dual immunofluorescence staining, identified infiltrating M1 macrophages as a major source of cardiac IL-6 101; elevated IL-6 in turn activates the cardiomyocyte JAK2/STAT3 axis, drives oxidative stress, and reinforces a feed-forward loop of M1 polarization and cytokine release 101. Because STAT3 is also a well-recognized pro-tumor effector downstream of IL-6 106, IL-6R blockade with tocilizumab is mechanistically attractive: it may preferentially restrain the IL-6–Th17 axis to dampen inflammation while largely preserving Th1 function and CD8⁺ cytotoxic activity, and simultaneously attenuate JAK2/STAT3-dependent tumor growth and metastatic signaling 101. Retrospective studies found that treating irAEs with anti-IL-6R therapy in melanoma patients increased the objective response rate from 56% to 68%, although evidence for the resolution of ICI-related myocarditis and its translation into clinical benefit remains limited 107.

In numerous autoimmune diseases, IL-23 drives the excessive proliferation and activation of inflammatory cells, thereby directly contributing to inflammation and tissue damage while playing a pivotal role in the underlying pathological processes of the disease 108. In the cohort of patients with irAEs, IL-23 levels increased with irAE severity and exhibited a monocyte-dominant expression profile in peripheral blood mononuclear cell transcriptomes, indicating upstream signaling driven by myeloid cells 100. Consistent with this, prophylactic IL-23 blockade effectively prevented myocarditis induced by dual CTLA-4 and PD-1 immunotherapy in humanized mouse models without compromising the antitumor efficacy of the combined immunotherapy 100. This suggests that IL-23 may serve as an intervenable node for decoupling therapeutic efficacy from toxicity. It is noteworthy that in ICI-related colitis, myeloid-derived IL-23 synergizes with the CXCL9/10–CXCR3 axis to expand Th17 cells and drive their shift towards a pathogenic IFN-γ⁺ IL-17⁺ dual-function state 109. This mechanism bears a resemblance to the CXCL9/CXCL10–CXCR3 positive feedback amplification circuit observed in ICI-related myocarditis in terms of its network architecture.

In summary, cytokine-targeted strategies offer a mechanistically grounded approach to disrupting the T cell–macrophage–driven inflammatory amplification loop in ICI-related myocarditis. However, the causal hierarchy and dominant amplification nodes among individual cytokines remain incompletely defined. Future studies integrating single-cell communication and spatial omics, particularly in models that better recapitulate the tumor–immune–cardiac microenvironment, will be essential. Clinically, a key challenge is whether cytokine blockade can mitigate cardiotoxicity without compromising antitumor efficacy, particularly in patients receiving combination ICI regimens. Addressing this trade-off will require prospective studies with paired cardiac endpoints and oncologic outcomes, rather than treating cardiac safety as an isolated objective.

### 3.4 The Inhibition of the IFN-γ-JAK-STAT1 Pathway

IFN-γ secreted by CD8^+^ T cells exacerbates ICI-related myocarditis through crosstalk with Cxcl9^+^Cxcl10^+^ macrophages, and the underlying mechanism has been described above. The findings from mouse and human studies support the potentially beneficial role of JAK inhibitors in ICI-related myocarditis. Specifically, *Ctla4*^+/-^*Pdcd1*^-/-^ mice exhibited significantly enhanced STAT1 expression and phosphorylation levels, while the use of the JAK1/2 inhibitor, ruxolitinib, significantly inhibited IFN-γ-induced Cxcl9 expression in BMDMs *in vitro*, suggesting that IFN-γ-JAK-STAT1 signaling plays an important role in the emergence of Cxcl9^+^Cxcl10^+^ macrophages 48. Bulk RNA-seq from ICI-related myocarditis patients also showed that JAK2 was the only gene in the JAK family that was significantly upregulated in ICI-related myocarditis patients compared to ICI-treated patients without evidence of myocarditis 110. There is growing evidence for the clinical efficacy of JAK inhibitors in a variety of autoimmune and inflammatory diseases, including autoimmune rheumatic diseases and inflammatory bowel diseases 111,112. Previous studies demonstrated that JAK inhibitors exhibit potential efficacy in the treatment of refractory ICI-associated colitis 113,114. Case reports have indicated that personalized JAK inhibitor therapy offers additional clinical benefit in corticosteroid-refractory ICI-related myocarditis, providing a novel option for intensified immunosuppression 115,116 **(Table 1).** Consistent with this, a recent multicenter observational study involving 24 patients with corticosteroid-resistant ICI-related myocarditis demonstrated a slight to moderate decline in cTnT levels after treatment with tofacitinib, a JAK1/3 inhibitor 117. However, among the five patients with life-threatening fulminant myocarditis who were unresponsive to both corticosteroids and tofacitinib, no sustained decrease in cTnT levels was observed 117. This indicates that tofacitinib may demonstrate differential efficacy across various forms of ICI-related myocarditis, particularly as an adjunctive therapy for patients who are either resistant to or have failed to successfully taper the corticosteroid treatment. However, in the critical group, tofacitinib alone may not be sufficient to control the disease, which may be related to the fact that tofacitinib primarily inhibits JAK1/3, rather than JAK2. However, tofacitinib demonstrated a favorable safety profile in terms of toxicity and antitumor activity in cancer patients who developed irAEs 117. Furthermore, in a cohort of 30 patients with ICI-related myocarditis who underwent a modified treatment regimen, the incorporation of systematic respiratory muscle screening and mechanical ventilation, along with high-dose abatacept and ruxolitinib therapy, significantly reduced the mortality associated with myotoxicity from 60% to 3.4% 110.

Overall, these preliminary studies suggest that JAK inhibitors may have potential benefits in the treatment of ICI-related myocarditis. However, further investigation into the mechanisms of the JAK pathway in this context, along with larger-scale prospective studies, is essential to confirm their efficacy and safety.

### 3.5 CTLA-4 Ig as a Promising Therapeutic Approach

Genetic studies have shown that *Ctla4* and *Pdcd1* interact in a gene dose-dependent manner to determine the frequency and severity of ICI-related myocarditis, while epidemiological evidence has also identified combined ICI therapy as a risk factor for this condition.

Abatacept, a CTLA-4-Fc fusion protein, interacts with the CD80/CD86 molecules on the surface of antigen-presenting cells, such as macrophages, effectively blocking the co-stimulatory signaling pathways essential for T cell activation 82,118. This mechanism differs from that of broad-spectrum immunosuppressants, potentially enabling a more targeted approach to mitigate ICI-related myocarditis, thereby reducing adverse effects. In the *Ctla4*^+/-^ *Pdcd1*^-/-^ murine model, treatment with abatacept resulted in a notable decrease in mortality and an improvement in cardiac pathology 82. Preliminary clinical evidence in the reported case series supports the use of CTLA-4 Ig for the treatment of ICI-related myocarditis, while abatacept may be an effective therapeutic option for severe and corticosteroid-refractory ICI-related myocarditis 82,119,120 **(Table 1)**. Concerns have been raised regarding the potential of CTLA-4 Ig, owing to its inhibitory effects on co-stimulation, to compromise the associated antitumor responses elicited by ICIs.

However, recent investigations have demonstrated that the co-administration of CTLA-4 Ig with ICIs may diminish antitumor efficacy, whereas its application after ICI therapy can enhance therapeutic outcomes 118. Post-immunotherapy treatment with CTLA-4 Ig does not significantly modify the expression of co-stimulatory molecules on APCs or influence the frequency or cytokine production of CD8^+^ T cells, suggesting that once CD8^+^ T cells achieve full activation following ICI therapy, continued co-stimulation may be less essential and does not hinder antitumor efficacy; rather, post-treatment CTLA-4 Ig enhances the therapeutic effect through a critical mechanism that involves reducing the frequency of Tregs 118. Moreover, Liu *et al.* evaluated belatacept and M17-2, which can bind to B7-1/B7-2 while failing to engage the clinically used anti-CTLA-4 antibodies, and these soluble CTLA-4 variants demonstrate superior efficacy in alleviating the severity of ICI-related myocarditis in murine models compared to abatacept, while concurrently preserving the antitumor efficacy of immunotherapy 121.

In summary, herein, we discussed the critical roles of autoreactive T cells, macrophages, and cytokines in the pathogenesis of ICI-related myocarditis. Hyperactivation of CD8^+^ T cells, especially in the absence of PD-1 and CTLA-4, leads to infiltration of autoreactive CD8^+^ T cells and macrophages, which triggers severe cardiac inflammation. Notably, the CXCL9/CXCL10-CXCR3 axis mediates interactions between T cells and macrophages, which exacerbate myocardial injury. Novel therapeutically targetable pathways, including targeting cytokines, JAK inhibitors, and CTLA-4, have shown potential to control myocarditis without significantly compromising antitumor efficacy. Consistent exploration of the potential mechanisms and emerging therapeutic options is essential to advance the treatment of ICI-related myocarditis and optimize patient prognosis.

## 4. Diagnosis and Prognosis of ICI-related Myocarditis

ICI-related myocarditis is characterized by an unpredictable time to onset and disproportionately high mortality rate, underscoring the critical need for early diagnosis and accurate risk stratification. Given the limited mechanistic clarity and the lack of targeted therapies, a proactive surveillance strategy that integrates biomarkers and multimodal imaging is essential for early detection and prognostic assessment.

### 4.1 Endomyocardial Biopsy (EMB)

EMB remains the histopathological gold standard for myocarditis diagnosis; however, its clinical utility in ICI-related myocarditis is constrained by sampling error, procedural risk, and the limited sensitivity of the Dallas criteria 122. ICI-related myocarditis is characterized by prominent CD68⁺ macrophage infiltration with abundant CD4⁺ and CD8⁺ T cells and myocardial PD-L1 upregulation 123. The incorporation of immunohistochemistry (CD3, CD8, and CD68) substantially improves diagnostic sensitivity compared with H&E staining alone 124. Moreover, pathological grading based on the inflammatory burden correlates with serum troponin levels and clinical outcomes, providing a potential prognostic value 125,126. Nevertheless, EMB is not recommended as a first-line diagnostic modality because of safety concerns and lack of evidence supporting its routine use 127.

### 4.2 Cardiovascular Magnetic Resonance (CMR)

CMR has emerged as the most informative noninvasive imaging modality for both the diagnosis and risk stratification of ICI-related myocarditis. In 2018, the updated version of the LLC was published, revising the diagnostic criteria for acute myocarditis to require the presence of at least one T2-based marker indicative of myocardial edema, and at least one T1-related marker reflecting myocardial damage (such as abnormal T1, extracellular volume, or LGE), in order to confirm the diagnosis of AM 128,129. Compared with other cardiac imaging modalities, CMR has a remarkable capacity for myocardial tissue characterization, rendering it a promising technique for identifying patients at high risk of ICI-related myocarditis.

A multicenter retrospective study encompassing 136 cases of ICI-related myocarditis revealed that 78% of patients exhibited abnormal T1 values, whereas 43% displayed altered T2 values 130. Thavendiranathan *et al.* employed the updated Lake Louise criteria and discovered that nearly all patients with ICI-related myocarditis met the diagnostic standards for non-ischemic myocardial injury 130. The LGE pattern observed in ICI-related myocarditis typically manifests as non-ischemic characteristics, predominantly localized to the mid-myocardial or epicardial regions, although it may also be present in the subendocardial layer or throughout the entire myocardial thickness 123. The most frequently affected areas include the anteroseptal, inferoseptal, inferior wall, and inferolateral wall 123. In over half of the patients with ICI-related myocarditis, the LVEF remains normal 131. Nonetheless, LGE remains a crucial marker for diagnosing ICI-related myocarditis via CMR imaging, although its sensitivity may be reduced when LVEF is normal and CMR is performed early 131.

### 4.3 Echocardiography

Echocardiography is the first-line imaging modality because of its accessibility and feasibility. While reductions in LVEF are uncommon, myocardial strain parameters, particularly global longitudinal strain and basal segment involvement, are frequently impaired and strongly associated with elevated troponin levels and major adverse cardiac events 132-134. Nonetheless, echocardiographic findings remain nonspecific and should be interpreted as supportive rather than diagnostic.

### 4.4 Positron Emission Tomography (PET)

Apart from suspected cases of cardiac sarcoidosis, 18F-fluorodeoxyglucose (FDG) is not routinely used in the clinical assessment of patients with myocarditis 135. Similarly, current international guidelines do not recommend PET for the diagnostic evaluation of ICI-related myocarditis 136. As a viable alternative for assessing myocardial inflammation, FDG-PET directly quantifies increased metabolic activity within the myocardial wall resulting from localized inflammatory cell activation 137. This approach provides complementary clinical insights alongside CMR findings indicative of myocardial edema in the context of diagnosing ICI-related myocarditis 138. Case reports have documented the ability of PET-CT to identify early-stage ICI-related myocarditis, particularly when CMR remains inconclusive 139-141. Furthermore, a retrospective, single-center study comparing CMR and ^68^Ga-DOTATOC PET/CT revealed the high sensitivity (100%) of the latter for detecting ICI-related myocarditis, especially during the early inflammatory stages where clinical symptoms are apparent; however, CMR remains unremarkable 142. PET also has the potential to detect concomitant myositis, thereby enhancing the comprehensive assessment of the disease extent.

### 4.5 Cardiac Troponin

Elevated troponin levels are a universal feature of ICI-related myocarditis 4,143,144. A retrospective cohort study indicated that all individuals diagnosed with ICI-related myocarditis presented with abnormal high-sensitivity troponin T (hsTnT) levels, suggesting that low hsTnT levels may exclude the diagnosis of ICI-related myocarditis 143. Nevertheless, the elevation of hsTnT levels warrants careful consideration, as it may be attributable to preexisting conditions or the direct cardiovascular effects of the underlying malignancy, rather than being a consequence of the ICI therapy itself 145. In this context, baseline troponin measurements before ICI treatment initiation are of significant value. Furthermore, analysis of 30 patients' troponin T (hs-TnT) levels before ICI treatment revealed that those with baseline hs-TnT levels ≥14 ng/L exhibited a heightened risk of cardiovascular events or progressive cardiac involvement within three months 146. Similarly, a retrospective analysis of 135 patients treated with pembrolizumab demonstrated that persistently elevated hs-TnI levels (>50 ng/L) at baseline and during follow-up were robust predictors of MACE, with baseline hs-TnI levels exhibiting a particularly strong correlation with all-cause mortality 147. Troponin I is the preferred biomarker for ICI-related myocarditis, because it more rapidly reflects myocardial injury in conditions such as myocarditis and exhibits greater specificity than creatine kinase (CK) and troponin T 148,149. However, subsequent research has indicated that cTnT may exhibit superior efficacy in assessing the risk of MACE. When cTnT levels exceeded 1.5 ng/ml, the risk of MACE escalated fourfold (HR 4.0; 95% CI 1.5 to 10.9; p = 0.003) 144. Similarly, in a prospective cohort study encompassing 60 patients with confirmed ICI-related myocarditis followed for one year, cTnT exhibited superior diagnostic sensitivity (98% vs. 88% for cTnI within 72 hours of admission) and a heightened predictive capacity for MACE within 90 days, with a cTnT upper reference limit ≥ 32 times identified as the potential predictive threshold 150. The optimal cardiac troponin thresholds for a clinically meaningful diagnosis and risk stratification remain elusive. Ischemic heart disease, akin to fulminant myocarditis, elicits pronounced elevation in cardiac troponin levels, thus necessitating the exclusion of acute coronary syndromes from differential diagnosis.

### 4.6 Non-cardiac Biomarkers

Furthermore, an observational cohort study revealed that patients with myocarditis frequently exhibit elevated levels of noncardiac biomarkers during treatment with ICI treatment, including alanine aminotransferase (ALT), aspartate aminotransferase (AST), lactate dehydrogenase (LDH), and creatine phosphokinase (CPK) 143. A multivariable analysis conducted by Alexi Vasbinder *et al.* identified that only CPK levels were significantly associated with the development of myocarditis (HR: 1.83; 95% CI: 1.59–2.10) and overall mortality (HR: 1.10; 95% CI: 1.01–1.20) 143. Notably, elevated CPK levels demonstrated a sensitivity of 99% and specificity of 23% in the identification of myocarditis 143.

## 5. Conclusions and Perspectives

In this review, we provide a comprehensive review of ICI-related myocarditis, describing the role of the heart in immune homeostasis and elucidating the complex interplay between autoreactive T cells, macrophages, and cytokines in the pathogenesis of this disease. Specifically, the development of ICI-related myocarditis is rooted in the intrinsic vulnerability of cardiac immune tolerance. Incomplete thymic presentation of cardiac TRAs allows autoreactive T cells to persist, and immune checkpoint blockade lowers the activation threshold of these clones, enabling their expansion, myocardial infiltration, and immune-mediated injury 16,82,84. In parallel, IFN-γ–driven inflammatory macrophage programs amplify CXCR3⁺ T-cell recruitment and activation, establishing a self-reinforcing macrophage and T cell inflammatory circuit that sustains myocardial inflammation and accelerates myocardial dysfunction 48,51. Despite increasing mechanistic clarity, several questions remain unresolved and should define the next phase of investigation. In particular, the developmental origin of pathogenic cardiac CD8⁺ T cells requires definitive elucidation. Dominant clonotypes may derive from naïve T cells primed within secondary lymphoid organs, from the expansion and functional reprogramming of pre-existing cardiac TRM cells, or from context-dependent contributions of both pathways 89. Moreover, the macrophage and T–cell amplification loop provides a framework for targeted immunomodulation: attenuating IFN-γ–JAK–STAT1 signaling or disrupting CXCL9/CXCL10–CXCR3 coupling may restrain upstream inflammatory propagation 48,51,110, whereas cytokine-directed strategies such as IL-17A or IL-23 blockade may reduce downstream tissue injury programs and, potentially, enable efficacy–toxicity decoupling 100,102. Critically, therapeutic development must maintain antitumor efficacy as its central constraint. These imperatives underscore the need for paired oncologic endpoints and rigorous consideration of treatment timing, dosing, and patient selection, rather than evaluating cardiac outcomes alone 100,117,118. Interpretation of mechanistic studies also requires caution. Many preclinical evidences are derived from mouse models, which are indispensable for pathway dissection and interventional testing 86,98,100,101, but cannot fully recapitulate the human immune environment or oncologic context. Accordingly, insights generated from experimental models should be validated in well-characterized patient cohorts, with confirmation in human tissue and clear links to clinically relevant outcomes. Looking forward, multi-omics strategies are positioned to resolve these gaps. Single-cell profiling has already revealed clinically relevant immune-state shifts in peripheral blood 85,151, while imaging and biomarkers remain central for diagnosis and risk stratification 130,144,150. Integrating single-cell RNA sequencing with spatially resolved assays in endomyocardial biopsies, complemented by single-cell TCR profiling, should connect clonal expansion to anatomical localization and *in situ* immune circuits 48,50. In parallel, blood multi-omics coupled with imaging phenotypes may help identify less invasive markers that predict onset, severity, and recurrence 123,143,150,151. Finally, given the diagnostic challenges and disproportionately high mortality associated with ICI–related myocarditis, multicenter cohort studies across diverse regions will be essential to delineate disease heterogeneity and to improve the generalizability of risk stratification frameworks and therapeutic pathways.

In conclusion, while ICIs have revolutionized cancer therapy, we must carefully navigate the challenges encountered along this path. As we strive to elucidate the mechanisms by which ICIs exacerbate cardiac inflammation, the development of targeted therapies that alleviate these adverse effects, without sacrificing the potent antitumor responses elicited by immunotherapy, remains the central focus of current research and clinical practice.

## Figures and Tables

**Table 1:** References for targeted treatment strategies in patients with ICI-related myocarditis

References (Year)	Study type	Number of patients treated	Targeted treatment strategy	Outcome evaluation	Conclusion
Salem, *et al.*^110^ (2023)	Single center, prospective, observational cohort study	30 patients treated with the modified strategy	Systematic screening and management of respiratory muscle involvement + mechanical ventilation when needed + high-dose abatacept + ruxolitinib + corticosteroids	Myotoxicity-related fatality decreased from 60% in the initial guideline-treated cohort to 3.4% (1/30) after implementation of the modified strategy.	Early recognition and management of respiratory muscle involvement, combined with CD86 receptor occupancy-guided abatacept and ruxolitinib, may reduce the high fatality rate of severe ICI-related myocarditis.
Liu, *et al.*^117^ (2024)	Multicenter, retrospective, observational study	53 irAE patients, including 48 with myocarditis	Tofacitinib-based immunosuppression, mostly 5 mg twice daily, with or without additional immunosuppressive therapy	Clinical remission rates were 54.5%, 96.7%, and 100% in the life-threatening, steroid-resistant, and steroid taper-failure groups, respectively.	Tofacitinib showed promising efficacy in irAEs, particularly in patients with steroid resistance or steroid taper failure; however, its efficacy may be limited in life-threatening fulminant myocarditis.
Xing, *et al.*^116^ (2022)	Case Report	1	Tofacitinib	Patient recovered clinically during the hospitalization without major adverse cardiac events.	Tofacitinib can offer additional clinical benefit in steroid-refractory cases and provides a new option for intensified immunosuppressive therapy in ICI-related myocarditis.
Nguyen, *et al.*^115^ (2022)	Case Report	1	Personalized-dose-adjusted abatacept + ruxolitinib + corticosteroids	Clinical improvement occurred within 7 days, with resolution of systolic cardiac dysfunction and ventricular arrhythmias, followed by successful discharge.	Personalized dose adapted immunosuppression using abatacept, ruxolitinib, and corticosteroids achieved quick reversal of fulminant, life-threatening ICI-related myocarditis refractory to standard therapy.
Salem, *et al.*^119^ (2019)	Case Report	1	Abatacept	Cardiac troponin T levels decline rapidly and ventricular arrhythmias resolve within 3 weeks.	Abatacept may be effective in the treatment of severe ICI-related myocarditis refractory to glucocorticoid therapy.
Liu, *et al.*^120^ (2020)	Case Report	1	Abatacept + mycophenolate mofetil + corticosteroids ± plasmapheresis	Heart failure symptoms improved, BNP decreased, and hsTnI stabilized and gradually decreased but remained persistently elevated.	Although aggressive multidrug combination immunosuppressive therapy (including Abatacept and MMF) may improve clinical symptoms, it may not completely resolve myocardial injury (as evidenced by persistent troponin elevation)

## Abbreviations

ICIs: immune checkpoint inhibitors
CTLA-4: Cytotoxic T-lymphocyte antigen 4
PD-1: Programmed death-1
PD-L1: Programmed cell death 1 ligand 1
irAEs: immune-related adverse events
scRNA-seq: Single-cell RNA sequencing
DP T cells: double positive T cells
pMHC: peptide-loaded major histocompatibility complex
cTECs: Cortical thymic epithelial cells
SP T cells: single-positive T cells
mTECs: medullary thymic epithelial cells
TRAs: tissue-restricted antigens
*Myh6*: the gene encoding α-myosin heavy chain
MYHCA: α-myosin heavy chain
PIA: post-infarction Autoimmunity
DCs: dendritic cells
TCR: T-cell receptor
Tcm: central memory T cells
Tem: effector memory T cells
Tscm: stem cell memory T cells
HGF: hepatocyte growth factor
EAM: experimental autoimmune myocarditis
RCMs: Resident cardiac macrophages
mAbs: monoclonal antibodies
PBMCs: peripheral blood mononuclear cells
CyTOF: time-of-flight mass cytometry
TRM: tissue resident memory
ANT1: adenine nucleotide translocase 1
KLF4: Krüppel-like factor 4
PNUTS: protein phosphatase 1 regulatory subunit 10
hBMSC-Exos: human bone marrow mesenchymal stem cells
